# High Humidity Leads to Loss of Infectious Influenza Virus from Simulated Coughs

**DOI:** 10.1371/journal.pone.0057485

**Published:** 2013-02-27

**Authors:** John D. Noti, Francoise M. Blachere, Cynthia M. McMillen, William G. Lindsley, Michael L. Kashon, Denzil R. Slaughter, Donald H. Beezhold

**Affiliations:** 1 Health Effects Laboratory Division (HELD), National Institute for Occupational Safety and Health (NIOSH), Centers for Disease Control and Prevention (CDC), Morgantown, West Virginia, United States of America; 2 Department of Microbiology, Immunology and Cell Biology, School of Medicine, West Virginia University, Morgantown, West Virginia, United States of America; University of Illinois at Chicago, United States of America

## Abstract

**Background:**

The role of relative humidity in the aerosol transmission of influenza was examined in a simulated examination room containing coughing and breathing manikins.

**Methods:**

Nebulized influenza was coughed into the examination room and Bioaerosol samplers collected size-fractionated aerosols (<1 µM, 1–4 µM, and >4 µM aerodynamic diameters) adjacent to the breathing manikin’s mouth and also at other locations within the room. At constant temperature, the RH was varied from 7–73% and infectivity was assessed by the viral plaque assay.

**Results:**

Total virus collected for 60 minutes retained 70.6–77.3% infectivity at relative humidity ≤23% but only 14.6–22.2% at relative humidity ≥43%. Analysis of the individual aerosol fractions showed a similar loss in infectivity among the fractions. Time interval analysis showed that most of the loss in infectivity within each aerosol fraction occurred 0–15 minutes after coughing. Thereafter, losses in infectivity continued up to 5 hours after coughing, however, the rate of decline at 45% relative humidity was not statistically different than that at 20% regardless of the aerosol fraction analyzed.

**Conclusion:**

At low relative humidity, influenza retains maximal infectivity and inactivation of the virus at higher relative humidity occurs rapidly after coughing. Although virus carried on aerosol particles <4 µM have the potential for remaining suspended in air currents longer and traveling further distances than those on larger particles, their rapid inactivation at high humidity tempers this concern. Maintaining indoor relative humidity >40% will significantly reduce the infectivity of aerosolized virus.

## Introduction

Winter influenza outbreaks occur with seasonal regularity in temperate climates and it has been suggested that humidity may affect transmission [1], [2]. Previous studies using influenza aerosols in small settling chambers generally concluded that aerosolized virus was inactivated at high relative humidity (RH) but survived much better at low RH [3], [4], [5]. Other studies [6], [7] revealed that survival was optimum at low RH, moderate at high RH and minimum at middle RH. The aerodynamic diameters of the aerosolized particles were not determined in any of these studies; therefore, the influence of particle size on inactivation of virus has not been reported. Lowen et al. [8] used a guinea pig model to directly test whether humidity affected aerosol transmission of influenza from infected animals to uninfected animals, housed in adjacent but separate cages in an environmental chamber with five RHs ranging from 20–80% at 20°C. In their study, transmission rates were 75–100% at 20%, 35%, and 65% RH, but only 25% at 50% RH and 0% at 80% RH. However, air samples were not collected to confirm that guinea pigs housed at different RHs shed similar amounts of aerosolized virus.

During the winter, people spend the majority of their time indoors and the risk of aerosol transmission of influenza by coughing, sneezing and breathing is a concern because respirable particles carrying influenza may remain airborne for prolonged periods. Influenza RNA has been detected in the exhaled breath and coughs of patients with influenza [9]–[11] and clinical studies during influenza seasons indicated that influenza was detected in aerosol particles ≤4 µm [12], [13]. A recent study of indoor locations where jet travelers are likely to interact with locals determined that RH is one of the primary factors associated with aerosol transmission of influenza [14].

Healthcare workers treating influenza patients are particularly prone to infection as they can be exposed to multiple patients in closed examination rooms over the course of a day. A novel approach to assess risk factors is the use of manikins in a controlled environment. This approach has been used to study the flow of human respired air in a room [15], the effects of ventilation on respired air [16]–[18], and the efficacy of surgical masks and respirators for protection of healthcare workers exposed to coughed influenza aerosols [19], [20].

To address whether humidity contributes to the risk of aerosol transmission of influenza, a simulated examination room equipped with environmental controls was constructed that contained a coughing and breathing manikin to simulate a healthcare worker’s exposure [19], [20]. In this study, the virus collected at the breathing manikin was separated into 3 size fractions according to their aerodynamic diameters (>4 µm, 1–4 µm, and <1 µm). We show that at low RH there is little loss in infectivity of virus from any particle fraction within the first hour but at moderate RH, 60–80% of the virus is inactivated and is dependent on viral particle size. The fastest rate of inactivation was seen in the >4 µm particle size where 78% of infectivity was reduced within 16–30 minutes of a cough.

## Materials and Methods

### Cells and Virus

Madin-Darby canine kidney (MDCK) cells (ATCC CCL-34) and Influenza strain A/WS/33 (H1N1, ATCC VR-825) were purchased from the American Type Culture Collection (ATCC, Manassas, VA) and maintained as described [21].

### Bioaerosol Samplers

National Institute for Occupational Safety and Health (NIOSH) bioaerosol samplers, which collect and size-fractionate aerosols into three fractions (>4 µm, 1–4 µm, and <1 µm aerodynamic diameters), were used to collect influenza-containing aerosols [12], [22].

### Real-time qPCR

The amount of total virus (infectious and non-infectious) in an aerosol sample was determined by real-time qPCR analysis to assess the number of Matrix1 gene copies as described [21].

### Viral Plaque Assay (VPA)

The number of infectious virus within an aerosol sample was determined by the VPA. Aerosols containing infectious influenza were inoculated onto a confluent lawn of MDCK cells and plaque forming units (PFU) were calculated as described [21].

### Aerosol Exposure Simulation Chamber

The simulated examination room (aerosol exposure simulation chamber) is 3.16 m×3.16 m×2.27 m high and includes a HEPA filter and an ultraviolet lamp [19], [20] to disinfect the chamber. The virus solution was aerosolized with an Aeroneb 2.5–4 µm micropump nebulizer (Aerogen, Galway, Ireland) and loaded into the cough simulator remotely for a total of 5 coughs at approximately 1 minute intervals as described [19], [20], [21]. The coughing simulator uses a metal bellows driven by a computer-controlled linear motor (Model STA2506, Copley Controls, Canton, MA) to reproduce the flow and aerosol pattern of a human cough. The cough had a 4.2 liter volume with a peak flow of 16.9 liters/second and a mean flow of 5.28 liters/second. The digital breathing simulator (Warwick Technologies Ltd., Warwick, UK) was equipped with a standard medium-sized head form (Sheffield model 189003, ISI Lawrenceville, GA). The breathing waveform was sinusoidal with a flow rate of 32 liters/minute (ISO standard for an adult 1.88 m tall with a mass of 85 kg engaged in moderate work) [23]. The coughing and breathing simulators were synchronized so that each cough was initiated at the start of an inhalation. NIOSH aerosol samplers collected aerosols 1 mm above the manikin’s mouth (through the mouth), 10 cm to the right and left of the mouth, and at two locations (P1 and P3) inside the room. For time course analysis, exam room air samples were collected from 3 samplers positioned outside the room (P2) to enable immediate processing of the collected samples. Aerosol particle concentrations in the exposure chamber were continuously monitored using an optical particle counter (OPC; Model 1.108, Grimm Technologies, Inc., Douglasville, GA) located 55 cm below the mouth of the coughing manikin The cough aerosol output from the cough simulator was measured using a Spraytec aerosol analyzer (Malvern Instruments, Worcestershire, UK).The aerosol exposure simulation chamber (Enviroline walk-in chamber, Norlake, Hudson, WI) maintained the selected temperature and humidity using a desiccant-based industrial dehumidifier (IAT-150-E, Innovative Air Technologies, Covington, GA), a centrifugal atomizer (Norlake), a remote heating/refrigeration system (NAWE150RL-3, Norlake) and a programmable temperature/humidity controller (CP8L, Norlake). After the chamber equilibrated at the desired temperature and humidity, the environmental control system was shut off and dampers within the system prevent aerosol particle losses in the dehumidifier and the heating/cooling air circulation system. The wall and floor seams of the chamber are sealed tightly with silicone caulk to prevent aerosol particles from leaking. The entrance door has manual locks that push the door tightly against seals that further prevent aerosol leakage during the equilibration and collection periods.

### Statistical Methods

The analysis of the number of PFUs induced by viral particles collected from the samplers was generated using SAS/STAT software, Version 9.2 of the SAS system for Windows (SAS Institute, Cary, NC). Data were transformed by calculating the natural log of PFUs prior to analysis to meet the assumptions of the statistical tests (homogeneity of variance). For samples collected for 60 minutes under 7 different RHs, a two-way factorial mixed-model analysis of variance (ANOVA) was performed on RH and fraction. This was done using RH as a numeric independent variable to calculate slopes, as well as a categorical variable to allow comparisons between mean levels of PFUs in each fraction at each RH. A significant interaction in a model with humidity as a numeric variable indicates that the slopes of the lines which plot PFUs as a function of RH are not equal across fractions. The second experiment, which sampled for 15 minute intervals for 60 minutes at 2 different RHs was analyzed with a three-way factorial mixed model ANOVA on RH, time and fraction, each being utilized as class variables. The final experiment which sampled for 60 minutes between hours 4 and 5 following aerosol generation was analyzed using a two-way mixed model ANOVA on RH and fraction. In all analyses, trial was included as a random variable in ‘Proc Mixed’ to account for the lack of independence between fractions in a given trial. Interactions were analyzed by examining simple main effects using the ‘slice’ option. All pairwise comparisons were considered significant at p<0.05.

## Results

### High Humidity Reduces the Infectivity of Influenza

**Figure 1:**
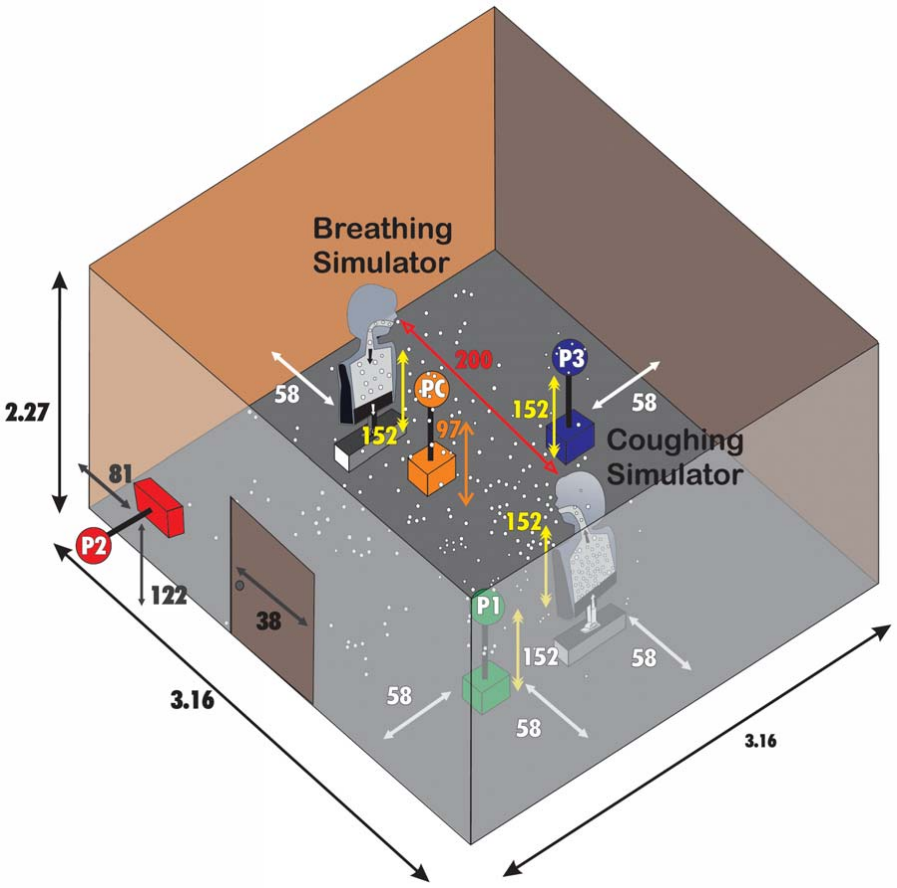
Three-dimensional view of the simulated examination room. National Institute of Occupational Safety and Health (NIOSH) samplers collected aerosols through the mouth, 10 cm on either side of the manikin’s mouth, and at 3 other positions (P1, P2, P3) as shown. The mouths of the coughing and breathing simulators and sampler inlets at P1, P2, and P3 were located 152 cm above the floor (approximate mouth height of a patient sitting on an examination table and a standing healthcare worker). All dimensions adjacent to white arrows within the room are in centimeters.

**Figure 2:**
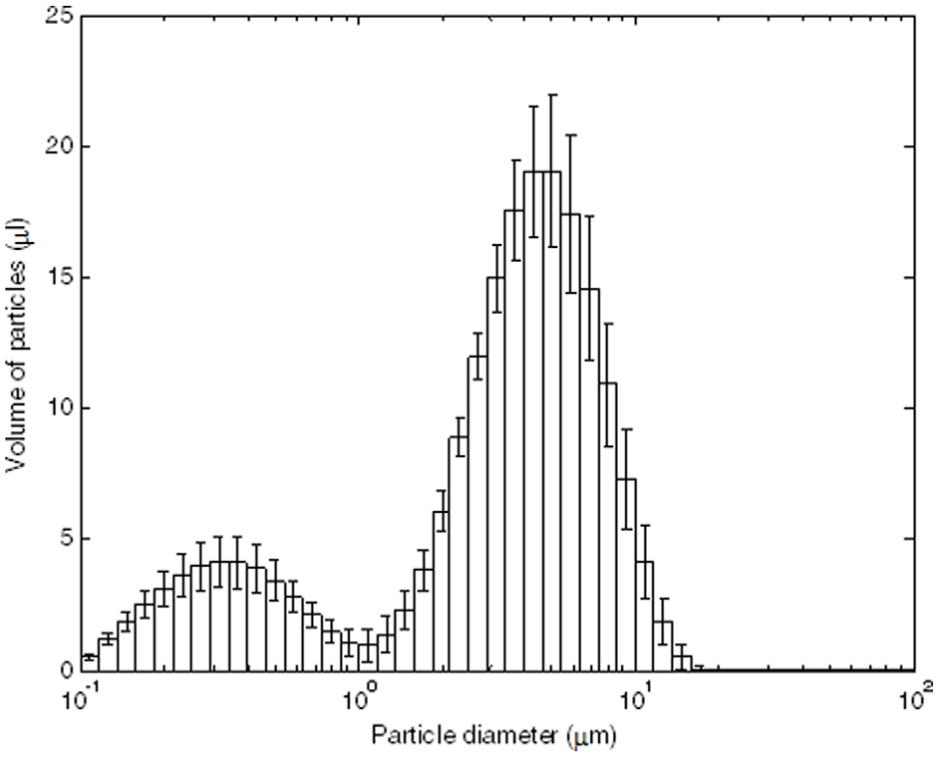
Cough aerosol particle optical size distribution. A particle counter was positioned just below the coughing manikin’s mouth. Each bar represents the total volume of the aerosol particles in that size range expelled during a single cough. The amount of virus in the particles is proportional to the aerosol volume. The plot shows the mean and standard deviation of 30 coughs (six sets of five coughs each performed as described in the Methods).

**Figure 3:**
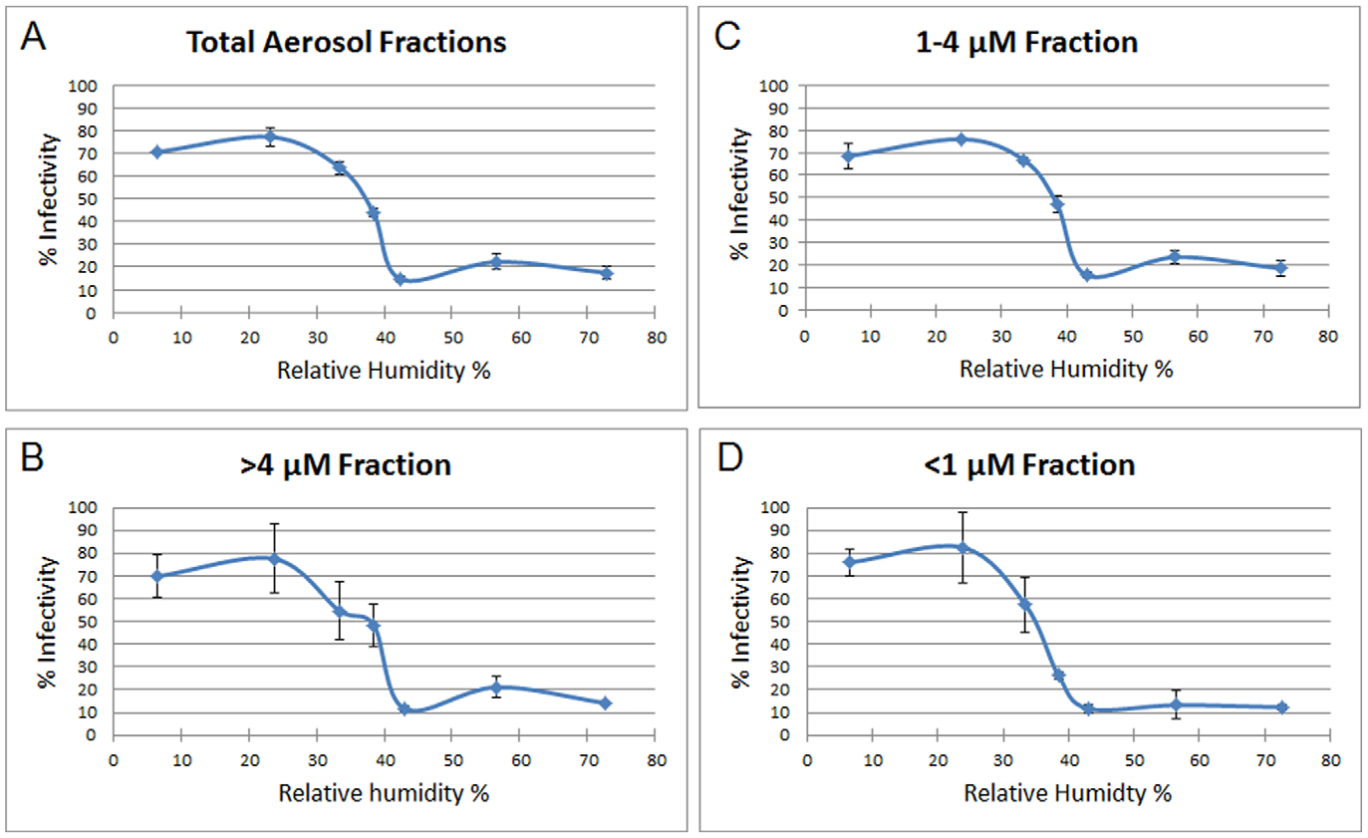
High humidity reduces the infectivity of influenza. Influenza virus was coughed into the examination room and NIOSH samplers collected aerosol samples for 60 minutes from the manikin’s mouth, 10 cm to the right and left of the mouth, and at positions P1 and P2 within the room. At constant temperature (20°C), the RH was varied over 7–73%.The percentage of virus that retained infectivity relative to that prior to coughing is shown. *A,* The percentage of infectious virus from all fractions (>4 µm, 1–4 µm, and <1 µm) was determined by the viral plaque assay (VPA) and is shown. *B–D,* The percentage of infectious virus within each aerosol fraction is shown. Data are means ± standard error (n = 5).

To assess the effect of humidity on infectivity, influenza virus was coughed into a simulated examination room where the RH was adjusted from 7–73%. The exam room contained coughing and breathing manikins facing each other and positioned 200 cm (∼6.56 ft) apart (Fig. 1). Approximately 1.0×10^8^ total virus was coughed into the exam room which equilibrated to 4.5×10^3^ total virus/per liter of room air (assessed by qPCR Matrix gene copies). A particle counter positioned just below the coughing manikin’s mouth showed that the coughed particles optical diameters were largely within the respirable size range (Fig. 2). Most of the virus was recovered in the 1–4 µm aerosol fraction (74.6% ± standard error 1.99%) and <1 µm fraction (18.5% ± standard error 2.17%); the remainder was detected in the >4 µm fraction (7.5% ± standard error 0.70%). The total amount of virus captured by each sampler was approximately the same regardless of their position within the room (data not shown). Approximately 4.6% of the 4.5×10^3^ total virus/per liter of room air loaded into the exam room was infectious prior to coughing (assessed by VPA). The percentage of virus that retained infectivity (number of PFUs/number of qPCR Matrix copies in an aerosol sample) relative to that prior to coughing was determined to be highest (70.6–77.2%) at 7–23% RH with a dramatic drop to the lowest (14.6%) at 43% RH (Fig. 3A). Increasing the RH to 57% resulted in a modest increase in the retention of infectivity (22.2%). A similar pattern of infectivity in response to humidity was observed among the three aerosol fractions when examined after 60 minutes of collection (Fig. 3B–D). Specifically, in each of the 3 fractions there was a significant decline in infectivity as humidity levels increased. However this percentage decrease in infectivity as a function of humidity occurs to similar extent across the 3 fractions as the 3 slopes are not significantly different from one another.

### Loss of Infectivity at Moderate Humidity Occurs Rapidly After Coughing

**Figure 4:**
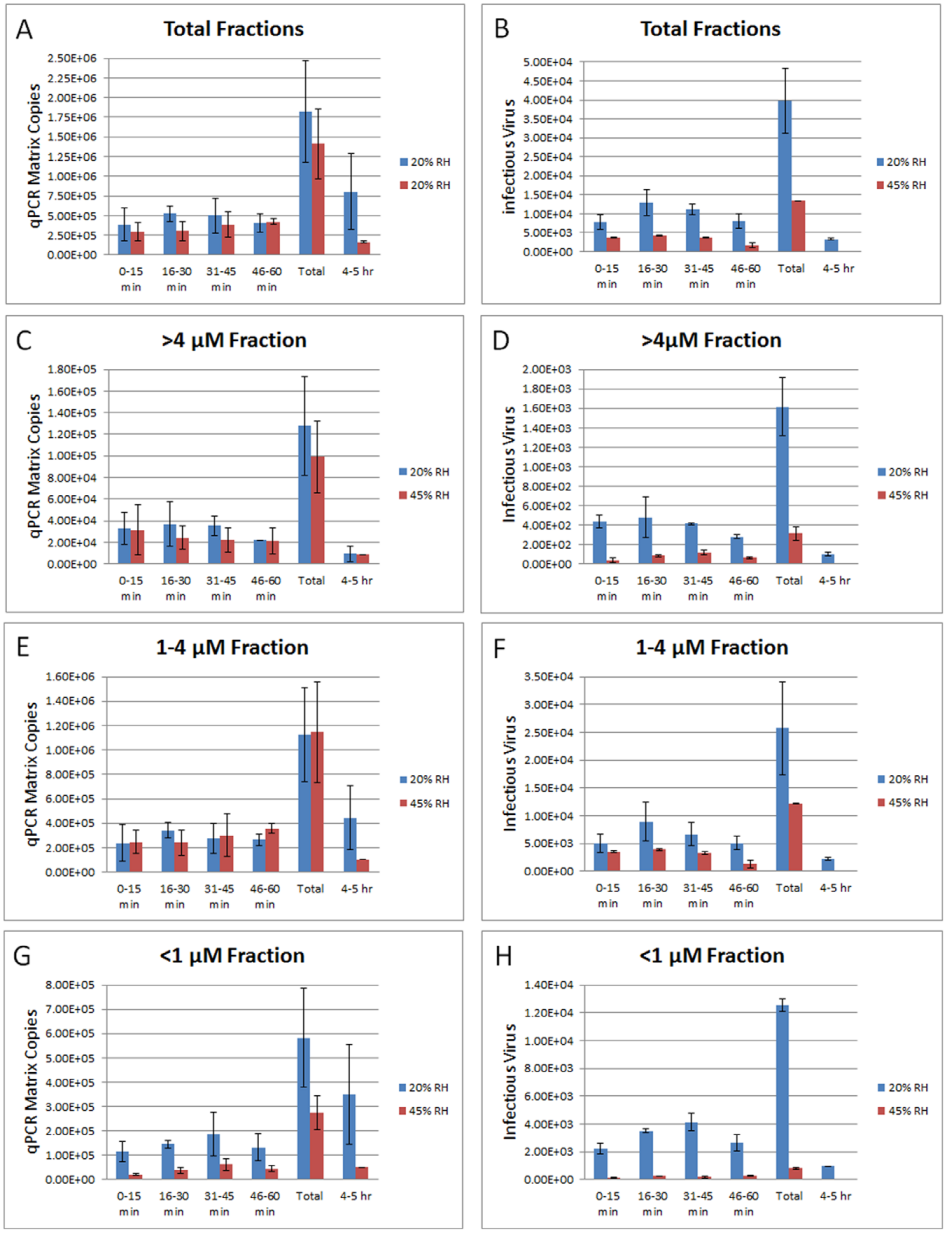
Loss of infectivity at moderate humidity occurs rapidly after coughing. Influenza virus was coughed into the examination room and NIOSH samplers collected aerosol samplers positioned on the outside wall of the examination room (P3) to enable immediate processing of the collected samples. Aerosol samples were collected at 16–30 min, 31–45 min, 46–60 min, and 4–5 h after coughing at 20% RH and 45% RH. The temperature of the examination room was maintained at 20°C throughout the collection periods. *A,C,E,G,* Amounts of total virus (infectious and noninfectious) from all aerosol fractions (>4 µm, 1–4 µm, and <1 µm) collected at each time interval was determined by quantitative polymerase chain reaction (qPCR). *B,D,F,H,* The number of infectious virus collected at each timepoint from all aerosol fractions was determined by viral plaque assay. The amount of virus collected at each 15 minute interval during the initial 60 minutes was totaled and shown as the “Total” on the X-axis of each graph. Data are means ± standard errors (n = 3 for each aerosol fraction assayed).

To determine how quickly aerosolized influenza is inactivated at increased RH, aerosol samples were collected at 5 intervals (0–15 min, 16–30 min, 31–45 min, 46–60 min and 4–5 h) after coughing and compared at 20% and 45% RH. The total amount of virus (assessed by qPCR of the matrix gene) collected during the initial 60 minutes after coughing was 1.8×10^6^ at 20% RH and 1.4×10^6^ at 45% RH (Fig. 4A). During this time, the total virus concentration within the exam room remained approximately the same throughout the 15 minute collection periods regardless of RH (Fig. 4A). Within the 0–15 min collection interval, 52% of the total infectious virus lost infectivity at 45% RH as compared to that found at 20% RH (Fig. 4B). Continued loss of viral infectivity occurred at each 15 min collection interval and at the later 4–5 h interval, however, loses were similar at both 20% RH and 45% RH (Fig. 4B).

### Aerosol Particle Size does not Confer Increased Stability of Influenza at Low RH

The amount of infectious virus present in the 3 aerosol fractions was then assessed to determine whether any one aerosol fraction carrying influenza virus retained infectivity longer at low RH. The amount of virus collected in the >4 µm aerosol fraction within the first 60 minutes of collection was approximately the same at 20% RH (1.3×10^5^ virus) and 45% RH (9.9×10^4^ virus) (Fig. 4C). Within the 0–15 min collection interval, >90% of the infectious virus in this fraction lost infectivity at 45% RH as compared to that found at 20% RH (Fig. 4D). Continued loss of viral infectivity occurred at each 15 min collection interval and at the later 4–5 h interval, however, loses were similar at both 20% RH and 45% RH (Fig. 4D).

The amount of virus collected in the 1–4 µm aerosol fraction within the first 60 minutes of collection was also approximately the same at 20% RH (1.1×10^6^ virus) and 45% RH (1.2×10^6^ virus) (Fig. 4E). Within 0–15 min after coughing, the loss in infectivity at 45% RH compared with that at 20% RH was not as high as that in the >4 µM fraction (29% loss vs >90% loss). However, as seen in the >4 µM fraction, there were continued losses in viral infectivity at each 15 min collection interval and at the later 4–5 h interval that were approximately the same at either 20% RH and 45% RH (Fig. 4F).

The amount of virus collected in the <1 µm aerosol fraction within the first 60 minutes of collection was more variable at 20% RH (5.8×10^5^ virus) then at 45% RH (2.7×10^5^ virus) (Fig. 4G). However, this 2–3 fold variability was consistent throughout the 15 minute collection intervals. Within 0–15 min after coughing, 94% of the virus within this fraction lost infectivity at 45% RH as compared to that at 20% RH (Fig. 4H). Continued loss of viral infectivity occurred at each 15 min collection interval and at the later 4–5 h interval, however, rates of loss were similar at both 20% RH and 45% RH (Fig. 4H).

Statistical analysis of the first 60 minutes showed there are significant main effects for humidity, fraction and time on virus infectivity and a significant humidity by fraction interaction. Specifically, with respect to humidity in general, infectious virus are reduced in the higher 45% humidity relative to low 20% humidity (p<0.0001). With respect to fraction, the number of infectious virus is highest in the 1–4 µM fraction and is significantly reduced in the <1 µM fraction and further reduced in the >4 µM fraction (p<0.0001). There was also a significant main effect of time (p<0.0068) with the first and last 15 minute collection intervals significantly lower than the two middle time points. The humidity by fraction interaction simply reflects that the size of the difference between the two humidity conditions varies as a function of fraction. Specifically, the smallest difference (while still statistically different) was in the 1–4 µM fraction while the largest difference in the number of infectious virus was in the <1 µM fraction. However, there was no statistical difference in the rate of decay of infectious virus at 20% RH versus that at 45% RH in any of the 3 aerosol fractions once the initial loss in infectivity occurred within 0–15 min after coughing.

## Discussion

The potential to transmit influenza by respirable aerosol particles (≤4 µm) is of particular concern as these particles can remain airborne for long periods and can be inhaled deeply into the lung to cause more severe infection [24]–[27]. Healthcare workers are at particular risk as they are directly exposed to the breaths and coughs of influenza patients which have been shown to contain virus [28], [29] and aerosolized virus has also been detected throughout clinic environments during flu seasons [12], [13]. The present study allowed us to assess viral infectivity under various levels of relative humidity and showed that one hour after coughing, ∼5 times more virus remains infectious at 7–23% RH than at ≥43% RH.

Yang and Marr [30] modeled the survival, size distributions, and dynamics of influenza emitted from a cough in an indoor environment and considered the roles of gravitational settling, ventilation, and virus inactivation at RHs of 10–90%. They concluded that settling can remove over 80% of airborne influenza 10 minutes after a cough and that RH increases the removal efficiency only slightly from 87% to 92% over the range of RHs. Applying a similar model to the cough aerosol particle distribution shown in Fig. 2, we estimated the change in the concentration of airborne particles in our chamber over time due to gravitational settling and filtration by the breathing simulator and aerosol samplers. We then predicted the amount of virus that should be collected in each stage of the aerosol sampler during the first hour (0 to 60 minutes) and the fifth hour (240 to 300 minutes) after the start of the series of coughs. Our results indicated that the amount of virus in the largest aerosol fraction (>4 µm) collected during the fifth hour would be reduced to 6% of that seen during the first hour; the second fraction (1–4 µm) would be reduced to 30%; and the smallest fraction (<1 µm) would be 58%. These model results compare very well to the actual viral particle collection results seen in Fig. 4B–D, where the amount of virus collected in each aerosol fraction during the fifth hour fell to 13%, 28% and 50% of the amounts detected during the first hour. The concentration of larger airborne particles decreases faster than smaller particles because larger particles settle much more quickly than smaller ones; in contrast, ventilation and filtration are not affected by particle size. Thus, settling accounts for much of the loss of particles >4 µm, whereas little settling occurred in the <1µm fraction.

Although most of the >4 µm particles were removed from the exam room at 4–5 h, a further decline in infectivity at 45% RH as compared to that at 20% RH nearly eliminates the potential for infection associated with particles of this size. Similarly, the potential for infection from influenza carried on the smaller particles was also further reduced at 45% RH, but the longer retention time of these particles in the air emphasizes the concern these sized particles still pose. The actual number of aerosolized viral particles that a healthcare worker could potentially inhale during a patient examination is largely dependent on the shedding rate of virus by the patient. Infected patients can shed 33 virus/min in aerosol particles ≥5 µm and 187 virus/min in particles <5 µm [28]. Therefore, in 30 minutes a single patient in a room the size of our simulated exam room can shed up to 5.6×10^3^ viral particles <5 µm in size and a healthcare worker could potentially inhale up to 237 viruses. A dose-response model developed by Teunis et al. [31] shows that the probability of infection by influenza is significant (P_inf_ = 0.2–0.4) at low doses (10^1–2^ TCID_50_ infectious units).

The effect of increasing humidity on viral survival differed among several reported studies as Hemmes et al. [3], Hood [4] and Harper [5] concluded that survival was maximum at 10–25% RH and minimal at high >50% RH whereas, Shechmeister [6] and Shaffer et al. [7] found survival was maximal at 20–25% RH, minimal at 50% RH, and moderate at 70–80% RH. High salt concentrations are deleterious to influenza [32] and protein concentrations in the viral preparation of less than 0.1 mg/ml adversely affect stability of influenza when aerosolized at high and mid-range RH [7]. Yang and Marr [31] suggest that, although Shechmeister [6] and Shaffer et al. [7] used significantly lower concentrations of protein in some of their viral preparations, the trends they obtained were the result of increasing salt concentrations followed by crystallization of the virus at the point of efflorescence (45–48% RH). In our study, 0.2% BSA was included to maintain stability of the virus, and our results support those obtained by Hemmes et al. [3], Hood [4] and Harper [5] and closely align with the Yang and Marr model.

Extrapolation of Harper’s [5] data of influenza aerosolized into a settling chamber over a range of RHs by Yang and Marr [31] revealed that infectivity of the total viral population is decreased faster at higher RHs and is evident 5 minutes after aerosolization. Our results indicate that the greatest effect of increased relative humidity occurs within 0–15 minutes after coughing and thereafter, the rates of inactivation of the virus within each aerosol fraction occurs at significantly slower rates regardless of humidity. Analysis of the aerosol fractions further indicates that the most rapid drop in infectivity within 0–15 min occurs in the >4 µM fraction (>90%) and that virus in the 1–4 µM fraction losses only 29% of infectivity during this time. Moreover, after correction for the lowered amount of virus detected by qPCR in the <1 µM fraction at 45% RH over that detected at 20% RH, the loss in infectivity during 0–15 min after coughing is ∼32%. Therefore, virus carried on smaller aerosol particles loose infectivity considerably slower. Yang and Marr [31] found that droplets shrink to one-half of their original diameter at 90% RH but to only two-fifths at 10% RH but whether droplet shrinkage accounts for these losses is unclear.

Hanley and Borup [14] examined aerosol transmission of influenza for indoor locations frequented by jet travelers and developed risk contours for temperature and humidity that were based on studies reported in the literature. They concluded that, in addition to intervention strategies including the use of masks and gloves, climate control of indoor locations should be considered by public health planners in making recommendations to interrupt the spread of influenza. The environmental controls in health care facilities are primarily designed to satisfy human comfort criteria established under ASRAE and ISO standards [33], [34] with the exception of special cases where higher humidity is specified to reduce static charge in medical test equipment and/or computer areas. Raising the humidity levels in existing facilities may not be practical given design limitations built into the facilities under the existing standards. However, if functional areas of health care facilities were identified as high risk for flu transmission due to low humidity conditions, consideration could be given during the design and construction phase of these facilities to accommodate maintaining appropriate recommended humidity levels.
